# *ezTrack*: An R Package for Accessible Exploration of Animal Tracking Data

**DOI:** 10.1002/ece3.74074

**Published:** 2026-08-05

**Authors:** Taylor B. Craft, Ruth A. Howison, Anne Beaulieu, Theunis Piersma, Mohamed Henriques

**Affiliations:** ^1^ Conservation Ecology Group, Groningen Institute for Evolutionary Life Sciences (GELIFES) University of Groningen Groningen the Netherlands; ^2^ BirdEyes‐Centre for Global Ecological Change University of Groningen Leeuwarden the Netherlands; ^3^ Knowledge Infrastructures Department, Campus Fryslân University of Groningen Groningen the Netherlands; ^4^ Department of Coastal Systems NIOZ Royal Netherlands Institute for Sea Research Den Burg the Netherlands

**Keywords:** black‐tailed godwits, data exploration, GPS tracking, home range, knowledge infrastructures, movement ecology, R programming, spatial ecology, telemetry

## Abstract

The use of tracking devices on wild animals has become widespread, providing unprecedented insight into the spatial and movement ecology of wildlife. However, translating tracking data into conservation‐relevant insight requires multiple data processing steps that can pose technical challenges for applied ecologists, students, and conservation practitioners. While several resources have been developed within the widely used R programming language to support detailed analyses of tracking data, their effective use often requires specialized spatial data‐wrangling skills. To encourage the responsible and reproducible handling of tracking data, and to improve the accessibility of movement‐data workflows, we introduce *ezTrack*, an R package designed to streamline common tasks in animal tracking data exploration. With a focus on simplicity, flexibility, and interoperability, *ezTrack* enables users to quickly import and standardize tracking datasets from a wide range of formats, summarize key tracking statistics, estimate home ranges, and generate clear visual outputs, including interactive maps. The package also produces rapid summary reports, supporting effective communication of tracking study results to stakeholders, collaborators, and decision‐makers. By offering an approachable tool for tracking data exploration and interpretation, *ezTrack* empowers a broader community of researchers, ecologists, conservation professionals, and students to engage with basic spatial ecology questions and extract useful information from their tracking datasets with limited technical input.

## Graphical abstract

This paper introduces an R package designed to make animal tracking data easier to clean, summarize, map, and visualize, especially for users who need practical workflows rather than highly specialized movement‐modeling tools. It provides functions for turning raw tracking data into a tidy format, generating summary statistics, plotting tracks and home ranges, and creating basic diagnostic visualizations.

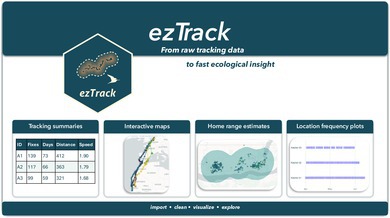

## 1 Introduction

Tracking devices on individual animals have been widely adopted in ecological and conservation research. Their use has changed research practices by enabling largely automated, high‐resolution movement tracking. Among other lines of research, the use of these technologies has been at the core of a number of novel and valuable insights into animal social behavior (Kays et al. 2015; Kempenaers and Valcu 2017; Lok et al. 2024), habitat selection (Byrne et al. 2014; Fieberg et al. 2021; Northrup et al. 2022), and migration patterns (Gill Jr. et al. 2009; Piersma et al. 2022; Das et al. 2025). Tracking devices in research therefore contribute a specific type of actionable information regarding species requirements and conservation planning for their key habitats (Katzner and Arlettaz 2020; Beal et al. 2025; Eren and Beaulieu 2025). Meanwhile, the growth of affordable and miniaturized tracking devices has contributed to a marked uptick of animal tracking datasets across taxa and ecosystems (Gould et al. 2024), from migratory birds (e.g., Brown et al. 2021; Zhu et al. 2021; Basso et al. 2023) to mammals (e.g., Byrne et al. 2014), and marine species (e.g., Voegeli et al. 2001).

Despite the increased production of tracking data, many challenges remain for ecologists and conservation professionals to efficiently and responsibly process and analyze these datasets. Raw tracking data are messy and cleaning them into usable standardized formats can be time‐consuming, particularly for those whose professional focus does not include working in programming environments, such as R (R Core Team 2024). Common tracking data exploration steps such as identifying time gaps, estimating utilization distributions, or even plotting animal locations often require the use of multiple packages or software tools, each with their own learning curve and dependencies. This fragmented workflow presents a barrier for many users, such as students, field biologists, and conservation project managers who need to communicate findings from their tracking studies in a timely manner without performing formal statistical analyses.

Several R packages provide sophisticated tools for movement ecology (for a comprehensive review, see Joo et al. 2020), but most assume familiarity with spatial data structures and statistical modeling frameworks. For example, *crawl* (Johnson et al. 2008) implements continuous‐time state‐space models to estimate animal trajectories from irregularly sampled tracking data. While this enables interpolation of positions and quantification of uncertainty, it requires model fitting and careful parameterization. Similarly, *ctmm* (Calabrese et al. 2016) extends this approach by explicitly modeling autocorrelation and irregular sampling intervals, producing more reliable home range and utilization distribution estimates through methods such as autocorrelated kernel density estimation.

Although statistically rigorous, these approaches can be challenging for users unfamiliar with likelihood‐based movement models and might constitute, in many cases, a poor effort‐benefit trade‐off for many users. The *amt* package (Signer et al. 2019) provides a structured workflow for step selection and resource selection analyses, focusing on habitat use while accounting for temporal dependence between successive steps. Despite its widespread adoption in applied ecology, it requires careful study design, semi‐regular sampling, and a solid grounding in statistical inference. In contrast, the conservation‐oriented *track2KBA* package (Beal et al. 2021) offers a streamlined framework for aggregating tracking data across individuals to identify candidate Key Biodiversity Areas (KBAs). It does this with deliberate emphasis on targeted conservation outcomes rather than general data exploration.

Visualization‐focused tools include *moveVis* (Schwalb‐Willmann et al. 2020), which generates animations of movement tracks aligned with remote sensing imagery. More general movement ecology workflows are supported by *move* (Kranstauber et al. 2022), which provides a comprehensive S4‐based infrastructure for managing and analyzing tracking data, including visualization, interpolation, segmentation, and integration with Movebank databases. Its successor, *move2* (Kranstauber et al. 2024), modernizes this framework by adopting the *sf* package (Pebesma 2018) and updating its geospatial dependencies.

While these packages are well‐suited for advanced analyses and hypothesis‐driven research, their use often requires navigating steep learning curves, including mastering S4 object systems, slot access (e.g., @ instead of $), and statistical modeling assumptions. For students, non‐specialist ecologists, and conservation practitioners, these can constitute barriers that limit adoption. Because R has become the *de facto* programming language taught in ecology and related fields (Lai et al. 2019), we start from the assumption that in practice, most people working with animal tracking data already possess at least a basic familiarity with R. Consequently, many users are neither complete beginners nor expert programmers, but instead fall between these extremes. This means they can take advantage of the reproducibility supported by script‐based workflows without relying on fully interactive or point‐and‐click applications. For this user group, the main aim is not complex modeling but quickly importing, cleaning, summarizing, and visualizing data in a reproducible way. To the best of our knowledge, no R package has been designed explicitly around exploration‐first workflows for non‐specialists that bring core tasks such as data import, cleaning, summary statistics, home range estimation, and visualization into a single, accessible interface.

To address this gap, we developed *ezTrack*, a lightweight and user‐friendly R package for importing, cleaning, summarizing, and visualizing animal tracking data. By streamlining these steps into one workflow, *ezTrack* enables users to focus on exploring their data and communicating results rather than navigating technical barriers.

## 2 Methods

### 2.1 Package Description

The suite of core functions of the *ezTrack* package (Table 1), available on the Comprehensive R Archive Network (CRAN), integrates widely used geospatial, movement ecology, and visual R libraries such as *sf* (Pebesma 2018), *geosphere* (Hijmans 2019), *adehabitatHR* (Calenge 2011), *ggplot2* (Wickham 2016), and *leaflet* (Cheng et al. 2023). In contrast, however, it places many of their complexities behind user‐friendly wrapper functions designed with animal tracking data in mind. The *ezTrack* package supports a standardized, reproducible pipeline that allows users to efficiently import, clean, summarize, visualize, and estimate home ranges from animal tracking data with minimal coding overhead. A full overview of the arguments available for each function is available in Table S1 in the Supporting Information, as well as directly in R through the help() function.

**TABLE 1:** Core functions available in *ezTrack*.

Function	Description	Purpose
*ez_track*	Import, standardize, and spatialize tracking data from a variety of common file types, including CSV, Excel, Shapefiles, GeoPackages, and GeoJSON	Allows the integration and comparison of datasets from diverse sources
*ez_summary*	Generate rapid summary reports of key tracking statistics	Helps users quickly assess data quality and coverage
*ez_fix_rate_plot*	Visualize individual location fixes over time	Provides visual overview of sampling effort and data gaps
*ez_map*	Visualize movement tracks and home ranges	Supports interactive exploration of animal space use and movements
*ez_home_range*	Estimate home ranges using either Minimum Convex Polygon (MCP) or Kernel Density Estimation (KDE)	Provides quick estimates of animal space use
*ez_latitude_plot*	Create plots of latitude over time	Enables detection of north–south movement patterns, such as migration and stopovers

### 2.2 Core Functions

The function *ez_track()* serves as the primary entry point for the *ezTrack* workflow. It accepts tracking data from a variety of common formats including CSV files, Excel spreadsheets, Shapefiles, and GeoPackages, as well as R objects such as data frames, data tables, sf, or Spatial class objects. The *ez_track()* function automatically detects the columns corresponding to individual identity, timestamp, and spatial coordinates, eliminating the need for manual specification in most cases. Appropriate column names are detected by indexing a list of possible combinations of common terminology (e.g., for individual identity: “individual”, “id”, “track”, “tag”, “device”, “name”). Although ez_track() automatically detects common column names in most cases, input datasets may still require manual variable assignment when column names are ambiguous. Users can therefore explicitly specify the columns corresponding to individual identity, timestamp, longitude, and latitude.

As a cleaning step, *ez_track()* parses timestamps, removes records with missing timestamps or coordinates, sorts locations by individual and timestamp, converts the data to a spatial object when requested, and removes duplicate records based on matching combinations of individual identities and timestamps. The cleaned data can be returned either as a standard data frame or as an sf object projected to WGS84. Data can also be temporally subsampled using the subsample argument, with intuitive commands such as “1 per day” or “2 per hour.”

To provide users with immediate insights into the quality and structure of their tracking data, *ezTrack* includes the function *ez_summary()*. This diagnostic tool calculates descriptive statistics for each tracked individual, offering a clear overview of the data coverage, potential gaps, and general movement characteristics. The function calculates the number of fixes, tracking duration, distance traveled, and average speed, among other summary statistics. Users can restrict these calculations to specific periods by filtering the data by date range directly within the function, using *start_date* and *end_date*. For quick reports, *ez_summary()* includes the option to generate an HTML‐formatted summary table that can be easily shared or added to presentations.

For a basic characterization of space use, *ezTrack* provides the *ez_home_range()* function, which implements two widely used methods for home range estimation: Minimum Convex Polygon (MCP) and Kernel Density Estimation (KDE). Both methods are implemented via the *adehabitatHR* package (Calenge 2011). The MCP method generates the smallest convex polygon that encloses a specified percentage of the location points, offering a straightforward geometric representation of the area used by an animal. In contrast, KDE produces smoothed probability surfaces that reflect the intensity of space use. Both methods are accessible within the same function call, with users selecting their preferred approach through the *method* argument, with the default being the MCP. The *level* argument defines the percentage of points to include in the home range calculation, with a default value of 95% but flexible to any user‐defined value. The function supports home range estimation at both individual and population levels, enabling a detailed look at single individuals, or aggregated estimates to inform broader patterns of shared space use. Results are returned as *sf* polygons, ready for mapping, further spatial analysis, or export to other dedicated GIS software. This function makes it possible to connect animal movement to specific conservation areas or jurisdictions, thereby providing a tool that enables data‐informed policy actions and documentation.

The visualization function, *ez_map()*, enables interactive exploration of tracking data. Built using the *leaflet* library (Cheng et al. 2023), this function provides an interface for plotting individual locations, movement paths, and home range polygons on interactive web maps. By default, *ez_map()* renders points and tracks colored by individual ID, with options to customize aesthetics such as marker size, line opacity, and color palettes. When home range polygons from *ez_home_range()* are used as input data, the function overlays these polygons on the same map, allowing users to visually compare movement trajectories and estimated space use. Because the maps are generated as interactive HTML widgets, they can also be easily embedded into R Markdown reports, websites, or exported as static maps.

The *ez_latitude_plot()* function visualizes time series of animal movements by generating plots of latitude values against timestamps. Built on the ggplot2 library (Wickham 2016), *ez_latitude_plot()* visualizes latitudinal shifts over time, providing insight into movement types like seasonal migrations or rhythmic north–south changes in space use across tracking periods. By default, the plot distinguishes individual animals using color, with options to facet the plot by individual for clearer separation when working with many animals. Users can further customize the appearance of the plot by adjusting date formatting on the *x*‐axis and specifying interval breaks for time labels. The function also supports date‐based filtering directly, enabling focused visualizations of specific time windows within the tracking dataset.

## 3 Case Study

To demonstrate the functionality of *ezTrack*, we present a case study using GPS and Argos PTT tracking data from a migratory waterbird species, the black‐tailed godwit ( *Limosa limosa limosa* , hereafter referred to as ‘godwit’). Within conservation projects such as the LIFE Godwit Flyway (2023) (https://www.godwit‐flyway.eu/), these tracking data are central to several project aims, particularly those focused on establishing baseline information on habitat use across understudied West African sites, and on identifying important areas on which to focus habitat restoration efforts. In this case study, we focus on the Gambia River, where very little is known about godwit habitat use. In such data‐poor contexts, even basic animal movement exploration can provide valuable guidance for conservation planning, such as identifying priority areas for wetland restoration.

The workflow begins by importing a tracking dataset. With a single call to *ez_track()*, datasets in common formats (.csv, .xlsx, .shp, or .gpkg) are cleaned, standardized, and converted into spatial objects. During this process, missing values and duplicate records are removed, column names are harmonized, and coordinates are projected to a consistent reference system.

We load a built‐in dataset of godwit GPS locations and pass it through *ez_track()*, producing a table as seen in Table 2.

**TABLE 2:** Preview of processed data from *ez_track()*.

ID	Timestamp	x	y
Tsjerkefeart	2020‐10‐16	−15.802	13.488
Tsjerkefeart	2020‐10‐18	−15.825	13.491
Tsjerkefeart	2020‐10‐23	−15.830	13.488

**Install development version of *ezTrack*:**
devtools::install_github("taylorbcraft/ezTrack")



**Install CRAN version of *ezTrack*:**
install.packages("ezTrack")



**Load and process data:**
library(ezTrack)data("godwit_tracks_gambia")godwit_ezTracks <‐ ez_track(godwit_tracks_gambia)head(godwit_ezTracks)



With the data standardized, the *ez_summary()* function produces individual‐level statistics, such as the number of fixes, duration of tracking, temporal gaps, and cumulative distance traveled (Table 3).

**TABLE 3:** Preview of summary statistics.

ID	First location	Last location	Duration (days)	Max time gap (days)	Locations per day
Abbegea	2016‐06‐16	2017‐11‐06	508	142	0.50
Adema	2023‐07‐11	2023‐11‐26	138	7	0.86
Aeltsjemar	2022‐07‐12	2023‐01‐02	174	8	0.91

**Calculate summary statistics:**
summary <‐ ez_summary(godwit_ezTracks)head(summary)



**FIGURE 1:**
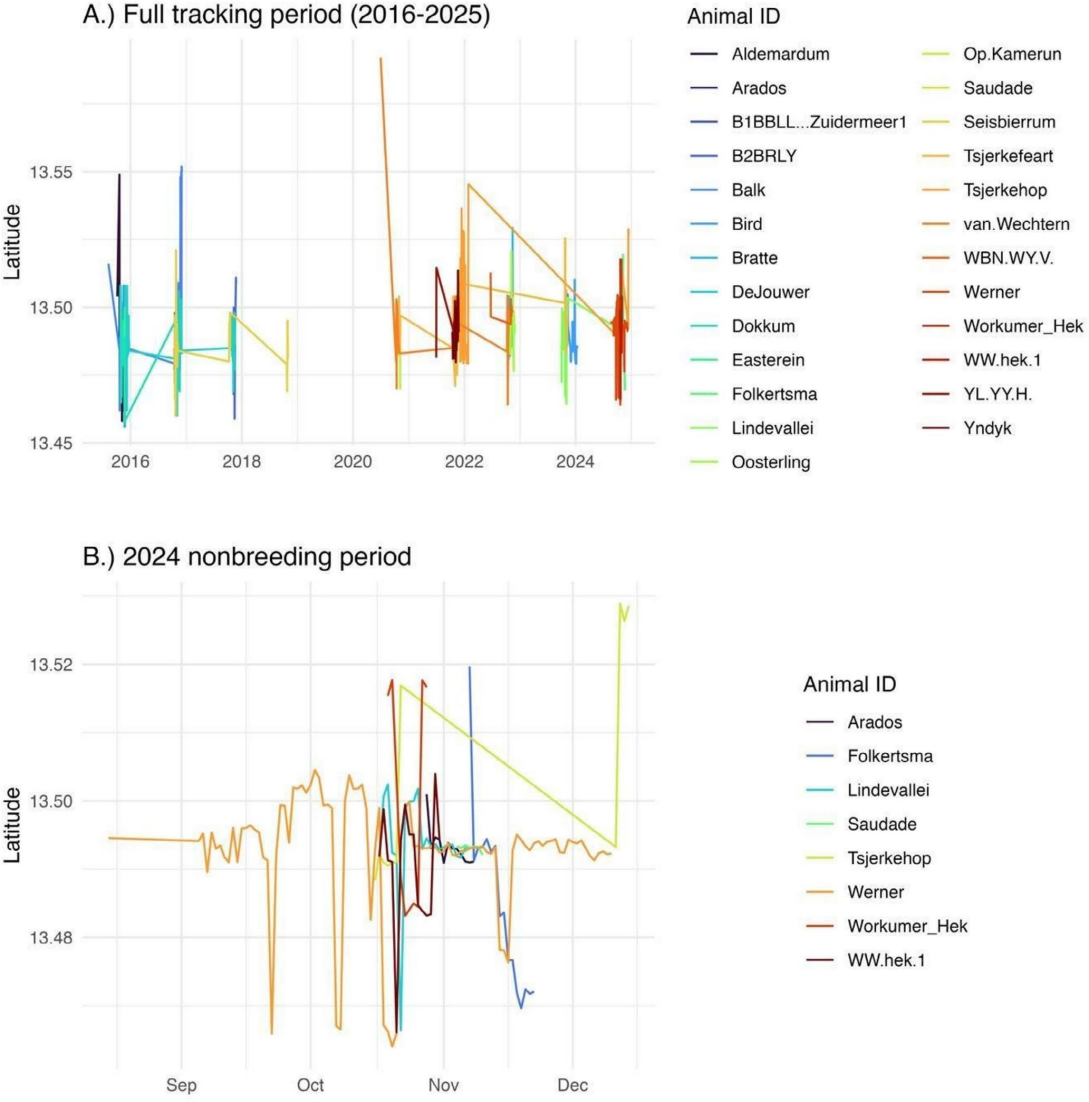
Latitude‐through‐time plot of black‐tailed godwit tracks in the Gambia generated with *ez_latitude_plot()*. Panel A shows the full tracking period, while Panel B shows locations filtered to the 2024 non‐breeding season. Each colored line represents an individual and shows changes in latitude across the tracking period, highlighting seasonal shifts in site use and timing of arrival and departure among years.

From here, exploratory visualizations can reveal broad‐scale movement patterns. The *ez_latitude_plot()* function provides a concise view of north–south movements over time, allowing users to identify seasonal shifts and periods of arrival or departure within a region, as shown in Figure 1.

**Create latitude plot for the full tracking period and the 2024 non‐breeding period:**
ez_latitude_plot(godwit_ezTracks)ez_latitude_plot(godwit_ezTracks,  start_date = "2024‐06‐01",  end_date = "2024‐12‐31")



**FIGURE 2:**
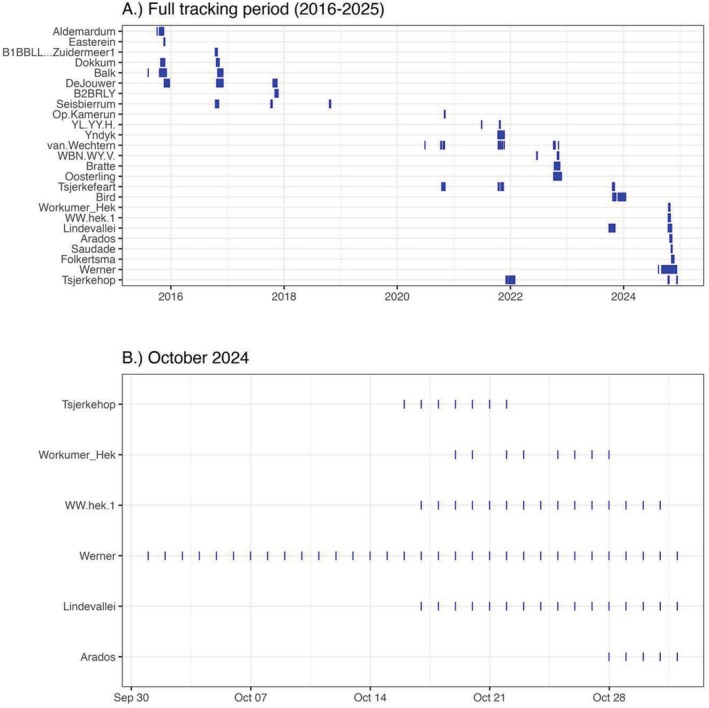
Temporal distribution of location fixes for individual black‐tailed godwits in the Gambia, generated with *ez_fix_rate_plot()*. Panel A shows the full tracking period from 2016 to 2025, while Panel B shows a subset of locations from October 2024. Each tick mark represents a recorded location, allowing rapid visual assessment of sampling effort, track duration, and gaps in data coverage among individuals and years.

Because data density can vary substantially across individuals and years, we examined the temporal distribution of location fixes with *ez_fix_rate_plot()*, which displays each location fix as a tick mark along the timeline (Figure 2).

**Create fix rate plot for the full tracking period and a shorter subset:**
ez_fix_rate_plot(godwit_ezTracks)ez_fix_rate_plot(godwit_ezTracks,   start_date = "2024‐10‐01",   end_date = "2024‐11‐01")



**FIGURE 3:**
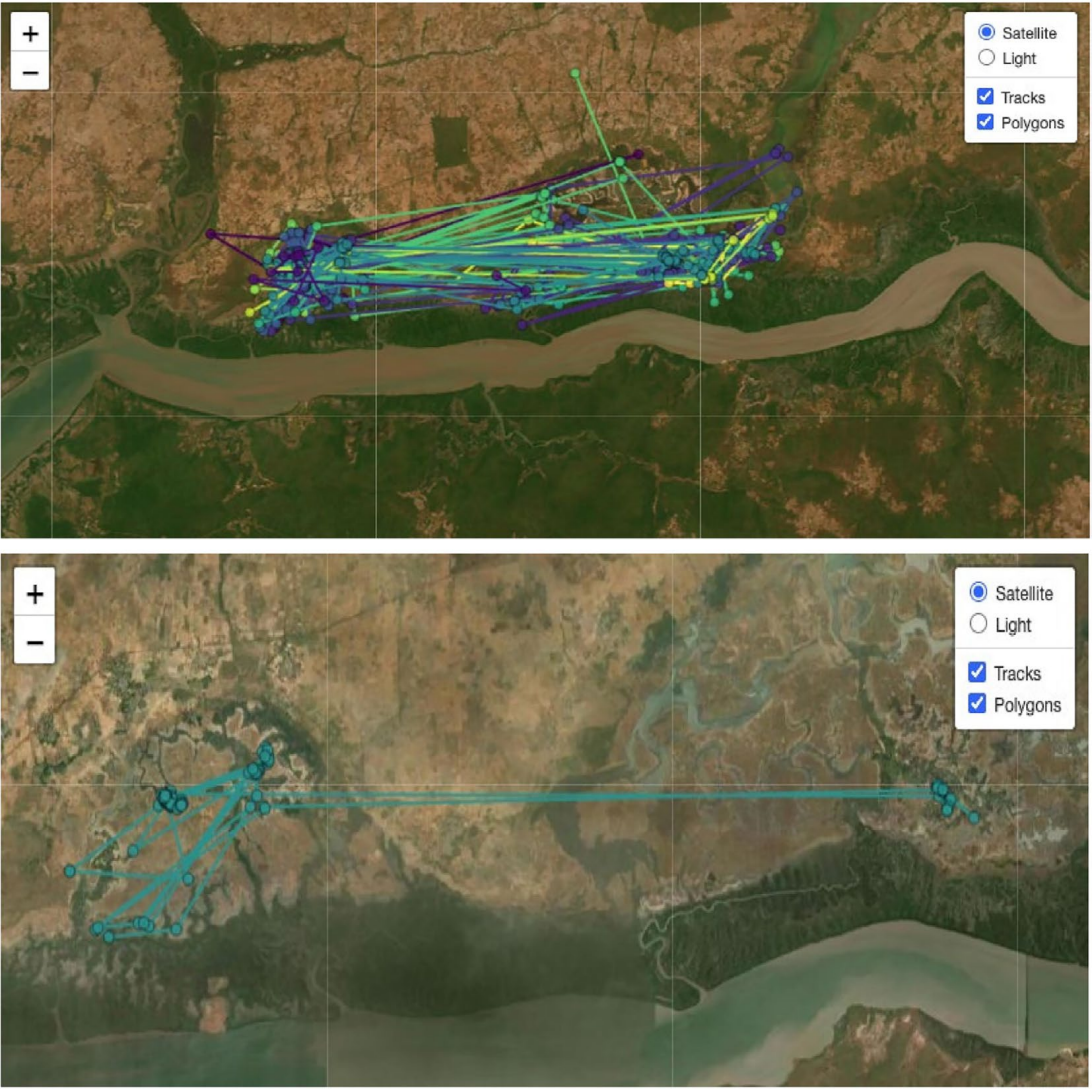
Interactive map of black‐tailed godwit tracking locations and movement paths in the Gambia generated with *ez_map()*. Colored points and line segments show individual records and trajectories over a satellite basemap, illustrating the spatial concentration of movements along the Gambia River. The top panel shows tracks for all individuals over 2015–2025, while the bottom panel shows tracks for a single individual, Werner.

Next, we visualized tracks with *ez_map()*, which combines tracks, points, and later, home range polygons on an interactive leaflet‐based map (Figure 3).

**Plot tracks for all individuals and a selected individual:**
ez_map(godwit_ezTracks)ez_map(godwit_ezTracks,  individual = "Werner")



**FIGURE 4:**
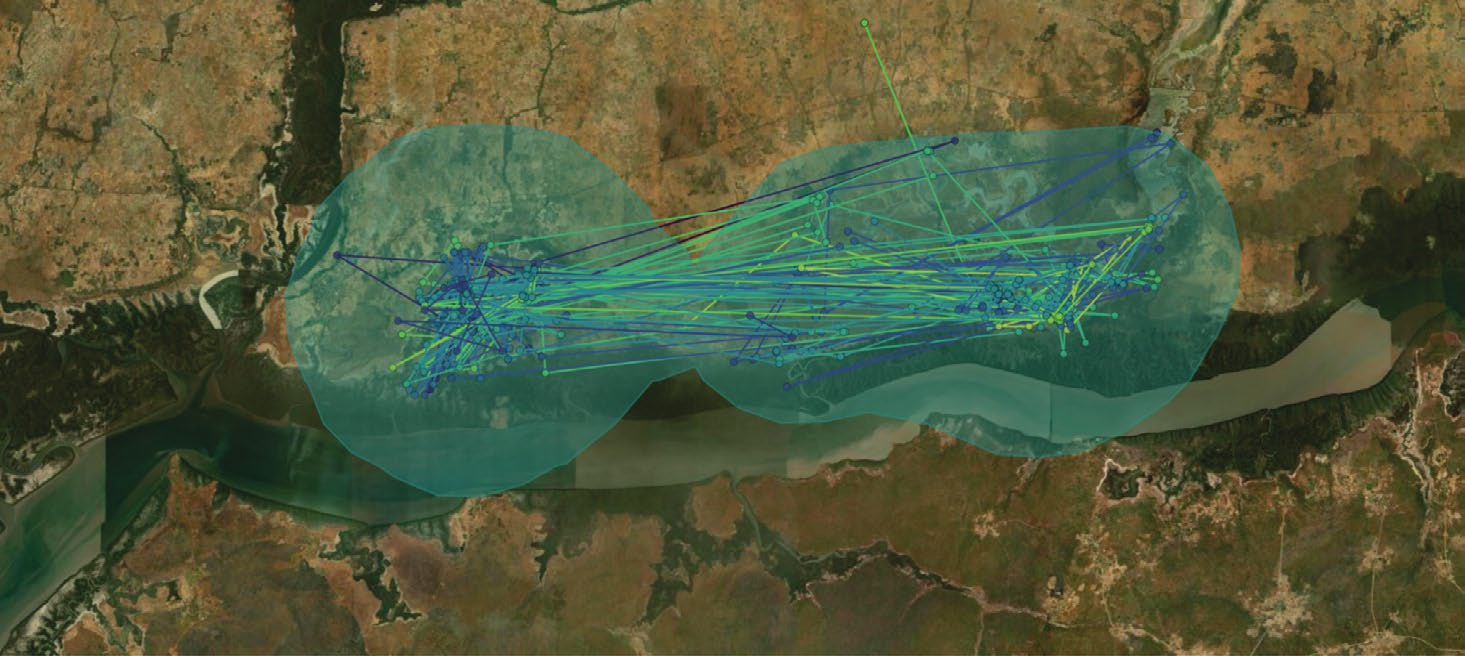
Population‐level kernel density estimate of black‐tailed godwit space use in the Gambia, generated with e*z_home_range()* and visualized with *ez_map()*. The shaded polygon shows the aggregated utilization distribution, identifying core areas of repeated use along the Gambia River.

Finally, we explored space use. The *ez_home_range()* function implements widely used methods such as Minimum Convex Polygon (MCP) and Kernel Density Estimation (KDE) via the *adehabitatHR* package (Calenge 2011). These home range polygons can then be visualized with *ez_map()* (Figure 4).

**Calculate and plot home ranges:**
home_range <‐ ez_home_range(godwit_ezTracks, method = "kde", population = TRUE)ez_map(home_ranges = home_range)



## 4 Discussion

The introduction of this R package constitutes a novel, accessible way to explore, clean, summarize, and visualize animal tracking data. This tool distinguishes itself from the current existing packages by focusing on offering a solid and simple workflow, tailored to users who aim to interact with their data in a straightforward and visual way without recourse to complex data processing skills. The primary functions of *ezTrack* require minimal parameterization and are designed to be plug‐and‐play for most tracking datasets. Default settings are provided to ensure immediate usability, while users also have the flexibility to adjust parameters if desired. This effectively enables users to focus on interpretation rather than technical implementation. Such an approach aims to facilitate ecological interpretation towards evidence‐informed decision‐making in conservation.

### 4.1 Comparison With Existing Movement Ecology Tools

In this paper we demonstrate the ease of use of *ezTrack* through a step‐by‐step description of the functions, and a case study analysis focused on analyzing a data set for mapping important areas for black‐tailed godwits in West Africa. Reaching the same conclusions with existing movement ecology tools would require substantially greater technical effort and more fragmented workflows, as illustrated by a more conventional process presented alongside the *ezTrack* approach in Code S1. Data import, cleaning, and standardization typically involve multiple object classes and packages, often relying on S4‐based infrastructures and manual coercion steps. Identifying individuals with insufficient data coverage, diagnosing temporal gaps, or assessing variable fix rates generally requires custom‐written code rather than dedicated functions. Exploratory visualizations, such as latitude‐through‐time plots to detect seasonal movements, must likewise be built from scratch using general plotting tools.

Home range estimates, which are extremely valuable for conservation planning (Schofield et al. 2013), often require chaining together multiple spatial packages, followed by additional work to visualize results on different basemaps. Although home range estimation methods are deeply nuanced, involving careful decisions about theoretical approaches, scale, projection, and ecological assumptions (Laver and Kelly 2010; Silva et al. 2021), not every project demands extensive customization or theoretical depth. In many cases, basic estimates are both sufficient and practical. However, as home range estimates can be affected by sampling resolution, outputs from ez_home_range() are best interpreted as rapid descriptive summaries rather than definitive estimates of space use. Responsible use of these outputs requires users to interpret them with a general understanding of the movement capacity of their study species and the structure of the underlying movement data.

While *ezTrack* is not intended to replace the analytical capabilities of more specialized and advanced animal movement packages, it offers a valuable complement to these tools by supporting the initial stages of data exploration, quality control, and spatial summarization. Its accessible design makes it well‐suited for classroom use, project development, workshops, field team reporting, and conservation practice settings where urgent issues must be addressed but where time and resources are limited. Researchers aiming to conduct detailed behavioral modeling, statistical hypothesis testing, or fine‐scale habitat analysis are encouraged to use *ezTrack* as a preparatory or complementary step that provides flexible data extraction parameters within broader analytical workflows. Although *ezTrack* performs routine preprocessing steps, other more specialized tools may be needed to model uncertainty or detect outliers. Users should therefore interpret outputs in relation to the biological context of their study system and inspect tracks carefully before using them in formal analyses and conservation reporting.

### 4.2 Case Study: Conservation Insight and Relevance

The case study provided illustrates how *ezTrack* supports simple but powerful animal tracking data exploration for conservation insight. In the case of godwits, it allowed us to move from raw tracking files to actionable knowledge, demonstrating not only that the Gambia River is used by godwits, but also when and where they concentrate. Such insights are essential for targeted conservation interventions in understudied regions, where baseline ecological information is scarce and spatially explicit evidence (e.g., maps) can be sufficient to create momentum for conservation planning. Such evidence‐gathering is particularly crucial for local agencies that are in a position to act and wish to do so based on evidence, but must also contend with limited access to specialized analytical tools or training.

Reports from *ez_summary()* provide rapid baseline information on godwit presence in the Gambia across years, while simultaneously identifying individuals that contribute sufficient data for spatial analysis versus those with sparse or fragmented tracks. This distinction is particularly valuable in long‐term tracking projects, where device performance, deployment conditions, and tag longevity often result in heterogeneous data quality that complicates downstream analyses. More generally, responsible presentation of tracking data requires users to communicate the limitations of the underlying data. The ez_summary() function supports this by providing summaries of data coverage that help users assess whether the data are suitable for a given interpretation. This is particularly important when outputs are used in conservation reporting, where visual summaries of animal movement can inform management decisions.

Temporal patterns of site use emerged clearly from exploratory visualizations. The *ez_latitude_plot()* revealed consistent movements of godwits into and out of the Gambia during the latter half of the year, allowing interannual comparison of arrival and departure timing. Complementarily, the *ez_fix_rate_plot()* highlighted periods of continuous tracking effort as well as extended gaps. These diagnostics provide essential context for interpreting spatial patterns, as well as for understanding tracking device location frequency over time. This type of output can also help with comparative assessments of tag performance, which can then be used to make informed choices when purchasing tags.

A population‐level KDE generated with *ez_home_range()* showed a concentrated pattern of space use along the north bank of the Gambia River, immediately adjacent to (but largely excluding) the mangrove belt. When visualized using *ez_map()*, these polygons delineated core areas repeatedly used by multiple individuals, providing a clear spatial basis for site prioritization. Notably, the tracks showed little to no use of rice cultivation areas in the Gambia, despite the well‐documented importance of rice fields for godwits elsewhere in their nonbreeding range (Lourenço and Piersma 2008; Wymenga and Zwarts 2010; Craft et al. 2025). These findings suggest conservation strategies that prioritize maintenance of natural wetland habitats and suggest that ongoing mangrove restoration initiatives in the region should be carefully targeted to avoid encroachment into core godwit sites. In addition, these outputs can guide field biologists in determining when surveys are most likely to encounter godwits, where ring reading efforts should be concentrated, and how to engage with local site managers and NGOs using clearly visualized, data‐driven criteria.

### 4.3 Outlook

*ezTrack* has been designed with the goal of empowering ecologists and conservation professionals to leverage the immense volume of tracking data made available within an increasing number of conservation‐oriented projects (Michel et al. 2026). Through this effort, we hope to make available a tool that increases the accessibility of complex analytical tasks by reshaping workflows into a set of simple steps that can lead to valuable and usable knowledge to support conservation action.

By lowering technical and computational barriers, *ezTrack* supports more equitable participation in the knowledge production of animal movement research, particularly for users in regions with limited access to computing infrastructure and advanced data analysis training. In this way, *ezTrack* contributes not only to the usability of tracking data but also to its distributivity (Renn 2021), fostering a broader and more diverse user base that includes students, field teams, and conservation actors operating in settings where proprietary tools and specialized training are not readily available. As a free and open‐source package, *ezTrack* promotes transparency, reproducibility, and global inclusivity in movement ecology and conservation research.

Future versions of *ezTrack* will expand functionality while preserving its simplicity. Planned enhancements include a lightweight S3 class system for improved extensibility, integration with remote sensing data for access to satellite imagery and environmental layers, and tools to incorporate landscape variables such as vegetation indices or land cover into movement analyses. Additional features will include spatial summaries of visits to user‐defined Areas of Interest (AOIs), home range overlap analysis, and automated outlier detection to support more efficient quality control and interpretation. Furthermore, we will also aim to integrate analytic outputs to assess temporal changes in movement patterns and space use, a critical knowledge type to understand the evolution of the conservation state of key biodiversity sites. Package maintenance will be conducted through GitHub, including automated checks to ensure the package builds and functions correctly. Users are encouraged to submit feedback or report issues via the GitHub issue tracker at: https://github.com/taylorbcraft/ezTrack/issues.

## Author Contributions

**Taylor B. Craft:** conceptualization (lead), data curation (lead), formal analysis (lead), investigation (lead), methodology (lead), project administration (lead), resources (lead), software (lead), validation (lead), visualization (lead), writing – original draft (lead), writing – review and editing (lead). **Ruth A. Howison:** conceptualization (supporting), funding acquisition (lead), investigation (supporting), methodology (supporting), project administration (supporting), supervision (lead), writing – original draft (equal), writing – review and editing (equal). **Anne Beaulieu:** conceptualization (supporting), project administration (supporting), supervision (supporting), writing – original draft (equal), writing – review and editing (equal). **Theunis Piersma:** conceptualization (supporting), funding acquisition (lead), investigation (supporting), project administration (lead), supervision (lead), writing – original draft (equal), writing – review and editing (equal). **Mohamed Henriques:** conceptualization (lead), formal analysis (supporting), investigation (supporting), methodology (supporting), project administration (lead), resources (supporting), supervision (lead), writing – original draft (equal), writing – review and editing (equal).

## Funding

This work was supported by LIFE Godwit Flyway, LIFE22‐NAT‐DE‐LIFE‐Godwit‐Flyway/101113618, LIFE IP GrassBirdHabitats, LIFE19 IPE/DE/000004.

## Conflicts of Interest

The authors declare no conflicts of interest.

## Supporting information


**Code S1.** *ezTrack* versus conventional workflow.

**Table S1:** Overview of possible arguments for each function in *ezTrack*.

## Data Availability

The package and associated data are publicly available through CRAN at https://cran.r‐project.org/package=ezTrack and via the package DOI: https://doi.org/10.32614/CRAN.package.ezTrack.
