# Supplementary material for: ezTrack: An R Package for Accessible Exploration of Animal Tracking Data
Source: Ecol Evol. 2026 Aug 5;16(8):e74074. doi: 10.1002/ece3.74074 (PMC13439628; doi:10.1002/ece3.74074)

Supplementary Code S1 – ezTrack versus conventional workflow

Data cleaning and conversion to spatial object

```
# ezTrack implementation:

tracks <- ez_track(godwit_tracks_gambia)
```

```
# Equivalent conventional workflow:

library(sf)
tracks <- godwit_tracks_gambia[, c("id", "timestamp", "x", "y")]

tracks$timestamp <- as.POSIXct(tracks$timestamp, tz = "UTC")

tracks <- tracks[!is.na(tracks$id) &
                                !is.na(tracks$timestamp) &
                                !is.na(tracks$x) &
                                !is.na(tracks$y), ]

tracks <- tracks[order(tracks$id, tracks$timestamp), ]

tracks <- tracks[!duplicated(paste(tracks$id, tracks$timestamp)), ]

tracks <- st_as_sf(tracks, coords = c("x","y"), crs = 4326)
```

Deployment timeline plot

```
# ezTrack implementation:

ez_fix_rate_plot(tracks)
```

```
# Equivalent conventional workflow:

library(ggplot2)
ranges <- aggregate(timestamp ~ id, data = tracks,
                    FUN = function(x) c(start = min(x), end = max(x)))
ranges <- do.call(data.frame, ranges)
names(ranges) <- c("id", "animal_timestamp_start", "animal_timestamp_end")

ranges$animal_timestamp_start <- as.POSIXct(ranges$animal_timestamp_start,
```

```r
                                           origin = "1970-01-01", tz = "UTC")
ranges$animal_timestamp_end <- as.POSIXct(ranges$animal_timestamp_end,
                                         origin = "1970-01-01", tz = "UTC")

ranges <- ranges[order(ranges$animal_timestamp_end, decreasing = TRUE), ]

tracks$y_labels <- factor(tracks$id, levels = ranges$id)
ranges$y_labels <- factor(ranges$id, levels = ranges$id)

ggplot(ranges, aes(x = animal_timestamp_start,
                   xend = animal_timestamp_end,
                   y = y_labels,
                   yend = y_labels)) +
  geom_segment(color = "black", linewidth = 0.8) +
  geom_point(data = tracks,
             inherit.aes = FALSE,
             aes(x = timestamp, y = y_labels),
             shape = 124,
             size = 3,
             color = "blue") +
  scale_x_datetime(date_breaks = "1 year",
                   date_labels = "%b %Y") +
  ylab("") +
  xlab("") +
  theme_bw() +
  theme(
    legend.position = "none",
    panel.grid.minor = element_blank()
  )
```

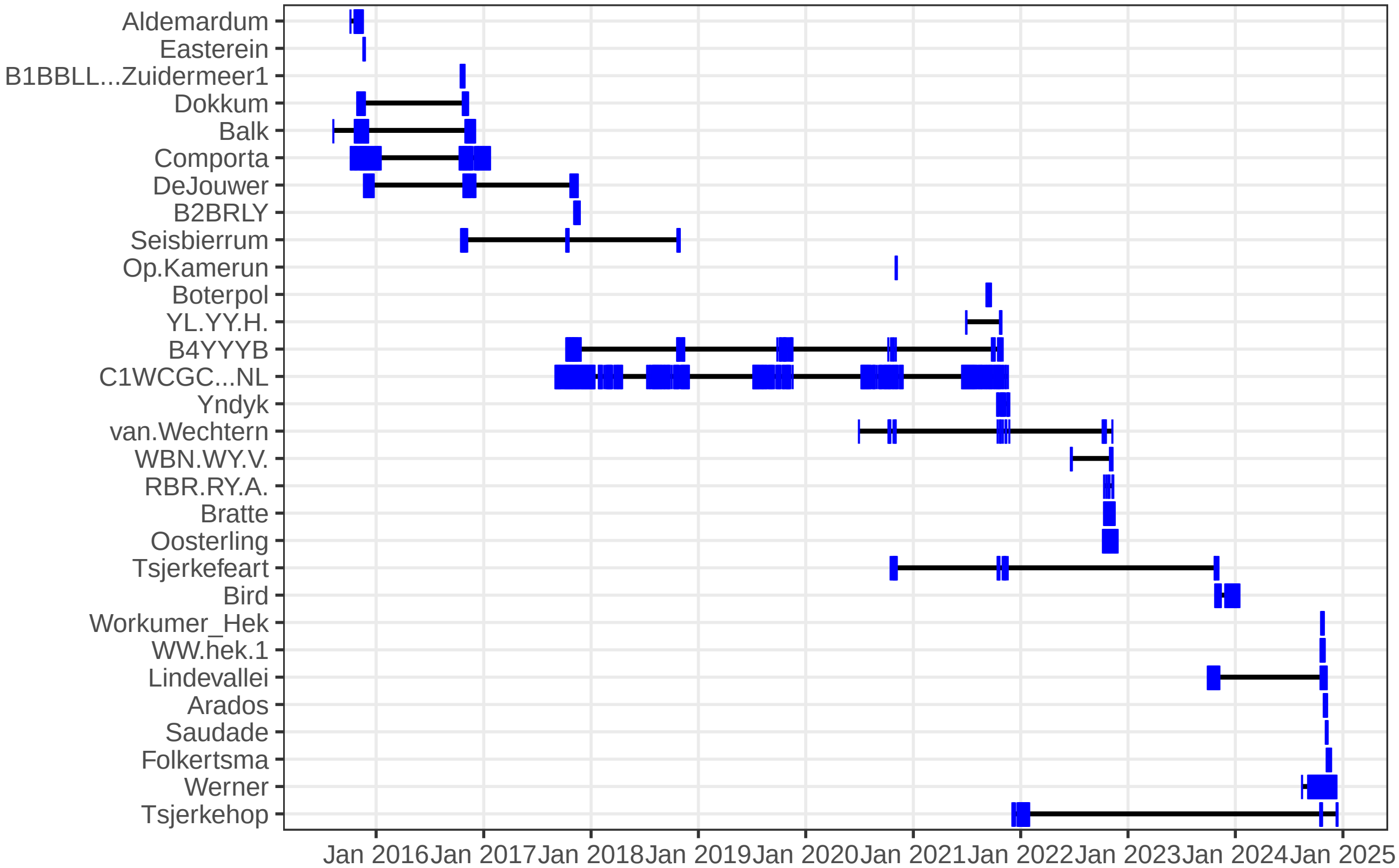

Home range estimation and plotting (95% KDE)

```r
# ezTrack implementation:

home_range <- ez_home_range(tracks, population = TRUE, method = "kde")
ez_map(home_ranges = home_range)
```

```r
# Equivalent conventional workflow:

library(sf)
library(adehabitatHR)
library(sp)
library(ggplot2)

godwit_tracks_sf <- st_as_sf(tracks, coords = c("x", "y"), crs = 4326)
godwit_tracks_utm <- st_transform(godwit_tracks_sf, 32628)

pop_sp <- as(godwit_tracks_utm, "Spatial")
pop_sp@data <- data.frame(group = factor(rep("population", nrow(tracks))))

kud_pop <- kernelUD(pop_sp[, "group"], h = "href", grid = 200)
kde95 <- getverticeshr(kud_pop, percent = 95)

kde95_sf <- st_as_sf(kde95)
```

```r
kde95_sf$level <- 95

ggplot() +
  geom_sf(data = kde95_sf, aes(fill = factor(level)), alpha = 0.25, color = NA) +
  geom_sf(data = godwit_tracks_utm, size = 0.6, alpha = 0.25) +
  labs(fill = "KDE level", x = NULL, y = NULL) +
  theme_bw() +
  theme(legend.position = "none")
```

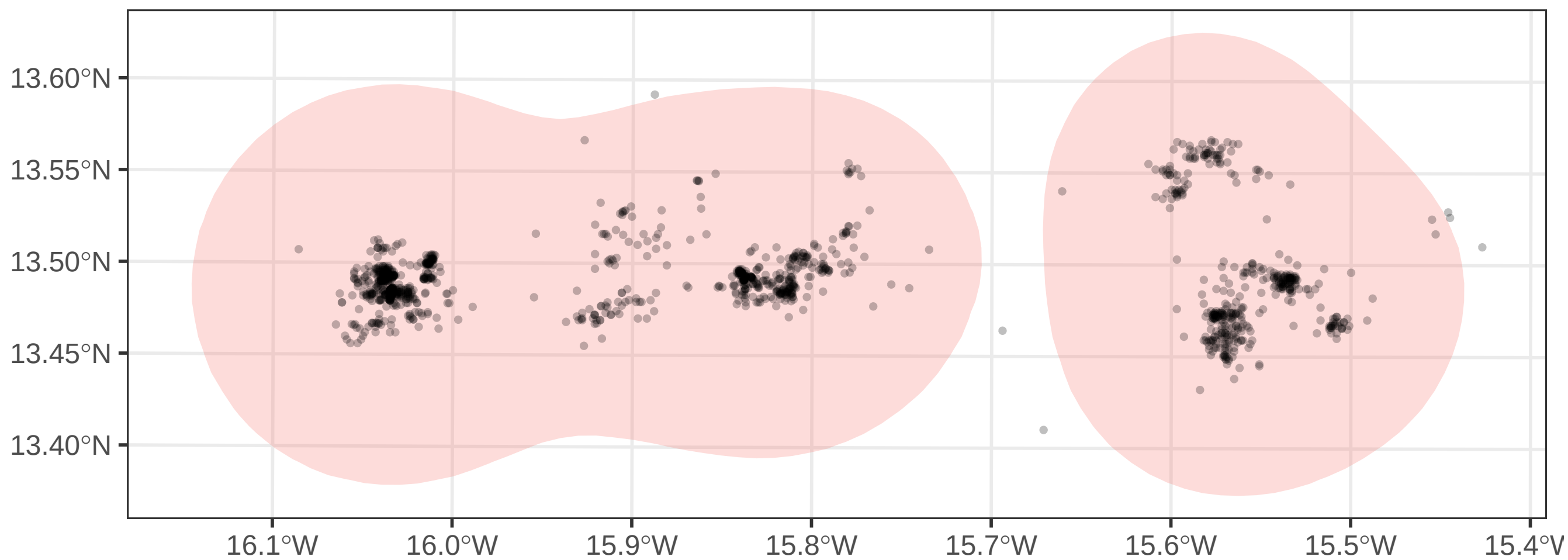

Supplement: Supplementary file 1 [file ECE3-16-e74074-s002.pdf]
