# Supplementary material for: ezTrack: An R Package for Accessible Exploration of Animal Tracking Data
Source: Ecol Evol. 2026 Aug 5;16(8):e74074. doi: 10.1002/ece3.74074 (PMC13439628; doi:10.1002/ece3.74074)
Supplement: Supplementary file 2 [file ECE3-16-e74074-s001.docx]

**Table S1**. Overview of possible arguments for each function in ***ezTrack***.

| Function | Argument | Default | Description |
| --- | --- | --- | --- |
| *ez_track* | data | – | A tracking dataset or file path. Accepted types: 'data.frame', 'sf', 'Spatial', or a path to CSV, XLSX, SHP, GPKG, or GeoJSON. |
|  | format | NULL | File format override (e.g., 'csv', 'xlsx'). |
|  | tz | "UTC" | Timezone for timestamps. |
|  | crs | 4326 | Coordinate Reference System of input data. |
|  | as_sf | TRUE | Return result as an `sf` object. |
|  | id | NULL | Column name for individual ID. |
|  | timestamp | NULL | Column name for timestamp. |
|  | x | NULL | Column name for longitude. If NULL, guessed automatically |
|  | y | NULL | Column name for latitude. If NULL, guessed automatically |
|  | keep_original_cols | TRUE | If FALSE, only retains 'id', 'timestamp', 'x', and 'y' |
|  | subsample |  | Specify how many fixes to keep per time unit (e.g.,"1 per hour" or "2 per day"). |
|  | verbose | TRUE | Print progress messages. |
|  | ... | – | Additional arguments passed to the read function. |
| *ez_summary* | data | – | Tracking data with columns `id`, `timestamp`, `x`, and `y`. |
|  | start_date | NULL | 'Date' object or string. Filters out data before this date. |
|  | end_date | NULL | 'Date' object or string. Filters out data after this date. |
|  | report | FALSE | Generate an HTML summary report. |
| *ez_fix_rate_plot* | data |  | A data frame or 'sf' object with columns 'id' and 'timestamp'. |
|  | date_breaks |  | Spacing of x-axis breaks. If 'NULL', ggplot2 chooses automatically. |
|  | date_format |  | Format string for x-axis date labels. If 'NULL', ggplot2 chooses automatically. |
|  | start_date |  | A 'Date' or string. Filters out fixes before this date. |
|  | end_date |  | A 'Date' or string. Filters out fixes after this date. |
| *ez_map* | tracks | – | Data frame or `sf` object with `id`, `timestamp`, `x`, and `y`. |
|  | home_ranges |  | An 'sf' polygon object |
|  | individual |  | Character or character vector of individual ID(s) to display. |
|  | show_paths | TRUE | Whether to draw path lines. |
|  | show_points | TRUE | Whether to draw points. |
|  | point_radius | 4 | Size of point markers. |
|  | point_color | "id" | Color points by column or value. |
|  | point_opacity | 0.8 | Fill opacity for points. |
|  | point_stroke | TRUE | Draw borders around points. |
|  | point_stroke_color | "black" | Color for point borders. |
|  | path_color | "id" | Color for path lines. |
|  | path_width | 2 | Line width for paths. |
|  | path_opacity | 1 | Opacity of path lines. |
|  | polygon_color | "id" | Column name or static color for home range polygons. |
|  | polygon_opacity | 0.4 | Fill opacity of polygons. |
|  | color_palette | "viridis" | Color palette to use for all mapped variables. |
|  | show_labels | TRUE | Show ID and timestamp on hover. |
|  | start_date | NULL | 'Date' object or string. Filters out data before this date. |
|  | end_date | NULL | 'Date' object or string. Filters out data after this date. |
| *ez_home_range* | data | – | Data frame or `sf` object with `id`, `timestamp`, `x`, and `y`. |
|  | method | "mcp" | Method: 'mcp' or 'kde'. |
|  | level | 95 | Percentage of points to include. |
|  | crs | NULL | CRS to project before calculation. |
|  | population | FALSE | Aggregate across individuals. |
|  | start_date | NULL | Filter: start date. |
|  | end_date | NULL | Filter: end date. |
|  | kde_extent | 1 | When method = "kde", passed to 'adehabitatHR::kernelUD()' to control the extent of the grid for KDE. |
|  | h | “href” | Bandwidth method when method = "kde". One of '"href"' (default), "LSCV", or a numeric value. |
|  | hlim | NULL | Optional vector of length 2 passed to 'adehabitatHR::kernelUD()' when method = "kde" to constrain the bandwidth search (used with h = "LSCV"). |
| *ez_latitude_plot* | data | – | Data frame or `sf` object with `id`, `timestamp`, `y`. |
|  | color_palette | "turbo" | Viridis palette option. |
|  | facet | FALSE | Separate panel per individual. |
|  | date_format | NULL | Date format for x-axis. |
|  | date_breaks | NULL | Interval for x-axis ticks. |
|  | start_date | NULL | 'Date' object or string. Filters out data before this date. |
|  | end_date | NULL | 'Date' object or string. Filters out data after this date. |
